# Recodon: Coalescent simulation of coding DNA sequences with recombination, migration and demography

**DOI:** 10.1186/1471-2105-8-458

**Published:** 2007-11-20

**Authors:** Miguel Arenas, David Posada

**Affiliations:** 1 Departamento de Bioquímica, Genética e Inmunología, Universidad de Vigo, 36310 Vigo, Spain

## Abstract

**Background:**

Coalescent simulations have proven very useful in many population genetics studies. In order to arrive to meaningful conclusions, it is important that these simulations resemble the process of molecular evolution as much as possible. To date, no single coalescent program is able to simulate codon sequences sampled from populations with recombination, migration and growth.

**Results:**

We introduce a new coalescent program, called *Recodon*, which is able to simulate samples of coding DNA sequences under complex scenarios in which several evolutionary forces can interact simultaneously (namely, recombination, migration and demography). The basic codon model implemented is an extension to the general time-reversible model of nucleotide substitution with a proportion of invariable sites and among-site rate variation. In addition, the program implements non-reversible processes and mixtures of different codon models.

**Conclusion:**

*Recodon *is a flexible tool for the simulation of coding DNA sequences under realistic evolutionary models. These simulations can be used to build parameter distributions for testing evolutionary hypotheses using experimental data. Recodon is written in C, can run in parallel, and is freely available from .

## Background

Coalescent theory [1] provides a very powerful framework for the simulation of samples of DNA sequences. Coalescent simulations can be very useful to understand the statistical properties of these samples under different evolutionary scenarios [2], to evaluate and compare different analytical methods [3], to estimate population parameters [4] and for hypothesis testing [5]. Not surprisingly, several simulation programs have recently been developed under this framework [6-12]. In order to obtain meaningful biological inferences from simulated data it is important that the generating models are as realistic as possible. However, increasing model complexity usually results in longer computing times, and most programs usually focus on a restricted set of biological scenarios. Currently, we lack a tool for the simulation of samples of coding sequences that have evolved in structured populations with recombination and fluctuating size, typical for example of fast evolving pathogens and MHC genes [13,14]. Here, we introduce a new simulation program, called *Recodon*, to fill this gap.

## Implementation

The simulation of data in *Recodon *is accomplished in two main steps. First, the genealogy of the sample is simulated under the coalescent framework with recombination, migration and demographics. Second, codon sequences are evolved along this genealogy according to a nucleotide or codon substitution model.

### Simulation of genealogies

For each replicate, genealogies are simulated according to thecoalescent under a neutral Wright-Fisher model [15,16]. Waiting times to a coalescence, recombination or migration event are exponentially distributed, and depend on the number of lineages, effective population size (*N*), recombination, migration and growth rates. Time is scaled in units of *2N *generations. Recombination occurs with the same probability between different sites (either nucleotides or codons). A finite island model [16,17] is assumed, where migration takes place at a constant rate between different demes. Multiple demographic periods can be specified, each one with its own initial and final effective population size, and length (number of generations). Positive or negative exponential growth is assumed.

### Simulation of nucleotide and codon sequences

*Recodon *implements several nucleotide and codon models that include different parameters (Table 1). The most complex nucleotide model implemented is the general time non-reversible model (GTnR; extended from Tavaré [18]), while the most general codon model is GY94∞GTnR_3∞4, which is the Goldman and Yang codon model [19], crossed with GTnR, and with codon frequencies predicted from the nucleotide frequencies at each codon position. Usually, the sequence at the root (most recent common ancestor or MRCA) is built according to the equilibrium frequencies, but the user has the option of specifying its own sequence. Note that in the presence of recombination, such sequence is just a concatenation of the MRCA sequences for the different recombinant fragments.

**Table 1:** Key arguments for Recodon. The user can specify several parameters to implement different simulation scenarios. These arguments can be entered in the command line or read from a text file.

Parameter	Example value	Application
Number of replicates	1000	All
Sample size	12	All
Number of sites (bp or codons)	3000	All
Effective population size	1000	All
Exponential growth rate	2.1 × 10^-5^	Demography
Demographic periods^1^	1000 5000 200	Demography
Recombination rate	5 × 10^-6^	Recombination
Migration rate	1.2 × 10^-4^	Migration
Number of demes	4	Migration
Mutation rate	5.1 × 10^-4^	All
Nucleotide frequencies^2^	0.4 0.3 0.1 0.2	Nuc/codon models
Transition/transversion ratio	2.1	Nuc/codon models
Relative substitution rates	1.0 2.3 2.1 3.0 4.2 1.0	Nuc/codon models
Nonsynonymous/synonymous rate ratio^3^	1.8	Codon models
Rate variation among sites^4^	0.5	Nuc/codon models
Proportion of invariable sites	0.2	Nuc/codon models

^1^from 1000 to 5000 effective size during 200 generations.

^2^can be specified for each codon position in codon models (3 × 4).

^3^*dN/dS*.

^4^shape of the gamma distribution.

### Program input

The input of the program consists of a series of arguments that can be entered in the command line or, more conveniently, specified in a text file (Table 1). These arguments fully parameterize the simulations, and control the amount of information that is sent to the console or output files.

### Program output

The principal output of the program is a set of sampled aligned nucleotide or codon sequences in sequential Phylip format. Additional information that can be saved to different files includes the genealogies, divergence times, breakpoint positions, or the ancestral sequences. Replicates can be filtered out depending on the number of recombination events, and an independent outgroup sequence can also be evolved. At the end of the simulations, a summary of the different events is printed to the console.

## Results and Discussion

We have developed a new program, called *Recodon*, for the simulation of coding DNA sequences. The program can run in parallel over multiple processors using the MPI libraries. The models implemented imitate the simultaneous action of several evolutionary processes, like recombination, migration, non-constant population size or selection at the molecular level. Understanding the joint effects of these processes is important in order to obtain more realistic estimates of population genetic parameters from real data [3,20-22].

### Program validation

*Recodon *has been validated in several ways. The output of the program was contrasted with the theoretical expectations for the mean and variances for different values, like the number of recombination and migration events, or the times to the most recent common ancestor [23]. In addition, results obtained with *Recodon *were in agreement with those obtained with other programs [10] under different evolutionary scenarios. Finally, substitution and codon model parameters were estimated from the simulated data using

HYPHY [24] and PAUP*[25]. The average parameter estimates from these programs agreed very well with the expected values from the simulations.

### Application

Coalescent simulations like those implemented in *Recodon *can be used to generate numerical expectations for different parameters under complex evolutionary scenarios, in which different processes interact in a simultaneous fashion. This can be very important to understand the interaction of different parameters, which complicates enormously their estimation [3]. Indeed, realistic simulation models are essential to evaluate different methods and strategies for estimating parameters and testing hypotheses from real data.

One potential application of *Recodon *could be the study of fast-evolving pathogens like HIV-1, which show high recombination and adaptation rates for coding genes [26]. For example, we could use this program to understand whether intrapatient genetic diversity for the *env *gene should increase with decreasing migration rates. Then we could test whether the number and diversity of *env *haplotypes sampled from a patient, all other conditions equal, ressemble the simulated cases with (or without) compartmentalization. Simulated data can also be used to obtain numerical estimates of population genetic parameter using approximate Bayesian computation [4,27-30]. Estimation by simulation can be especially useful in situations where the likelihood for a model is not known, or is computationally prohibitive to evaluate, which is often the case under complex biological scenarios.

**Figure 1:**
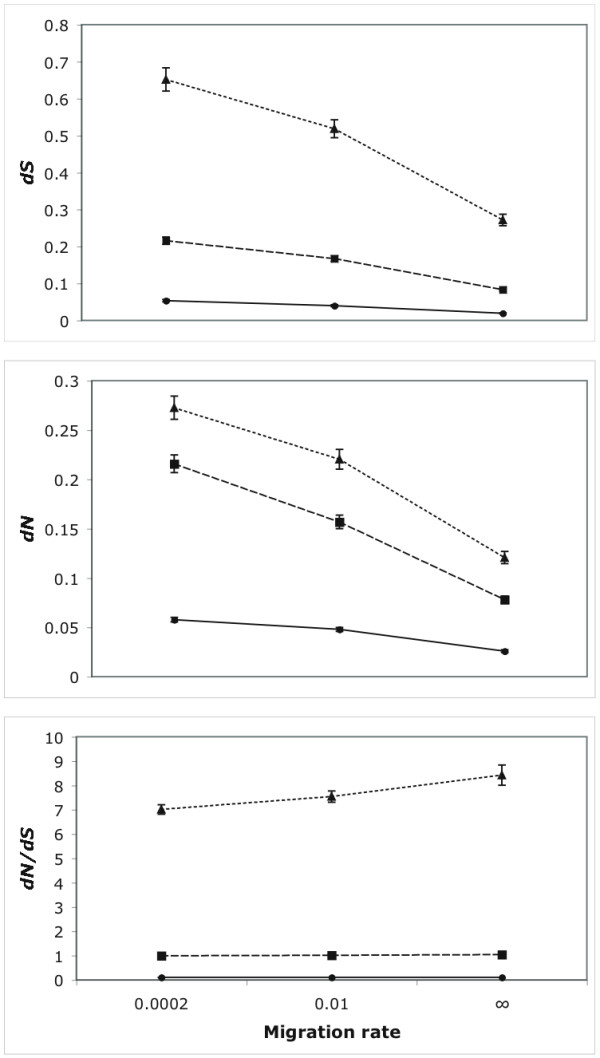
**Effect of population structure on the estimation of synonymous and nonsynonymous divergence**. Nine different scenarios were simulated, combining three migration rates (*m *= 0.0002, 0.01 and ∞ (= one deme)) and three *dN*/*dS *ratios (dashed line = 0.1, solid line = 1, dotted line = 10). For each scenario, 500 alignments with 10 sequences 333 codons long, were simulated. In all cases, the mutation rate was 5.4 ∞ 10^-5^, the transition/transversion ratio was 1.0, and the effective population size was 1000. Mean synonymous divergence per synonymous site (*dS*), nonsynonymous divergence per nonsynonymous site (*dN*), and their ratio (*dN*/*dS*) were estimated according to Nei and Gojobori [32] with a modified version of SNAP [33]. Error bars indicated approximate 95% confidence intervals (± s.e. ∞ 1.96).

In addition, we carried out a very simple experiment to illustrate another possible use of *Recodon*. In particular, we studied the effect of population structure on the footprint of molecular adaptation. Results suggest that population subdivision tends to increase both *dN *and *dS *divergences, as a result of longer times to the most recent common ancestor (Figure 1). This increase is similar in magnitude, and the *dN*/*dS *ratio is not affected by different migration rates when the simulated value is below one or one, but there seems to be a slight increase when the simulated *dN*/*dS *is 10.

### Future development

In the future we plan to relax some of the current assumptions, like an homogeneous recombination rate [31].

## Conclusion

*Recodon *is a versatile program for the simulation of codon alignments under complex population models. This program fills a gap in the current array of coalescent programs for the simulation of DNA sequences, as no single program is able to simulate codon sequences sampled from populations with recombination, migration and growth. Data simulated with this program can be used to study both theoretical and empirical properties of DNA samples under biologically realistic scenarios.

## Availability and requirements

*Recodon *is written in ANSI C, and it has been compiled without problems in Mac OS X, Linux Debian and Windows. It can run in parallel using the MPI libraries in architectures with several processors. The program is freely available at , including executables, source code and documentation. The program is distributed under the GNU GPL license.

## Authors' contributions

Recodon is an extension of a coalescent program written by DP, who conceived the idea and supervised its development. MA wrote and validated the program. Both authors drafted the manuscript, and both read and approved its final version.
